# Prognostic factors, therapeutic approaches, and distinct immunobiologic features in patients with primary mediastinal large B-cell lymphoma on long-term follow-up

**DOI:** 10.1038/s41408-020-0312-7

**Published:** 2020-05-04

**Authors:** Hui Zhou, Zijun Y. Xu-Monette, Ling Xiao, Paolo Strati, Fredrick B. Hagemeister, Yizi He, Huan Chen, Yajun Li, Ganiraju C. Manyam, Yong Li, Santiago Montes-Moreno, Miguel A. Piris, Ken H. Young

**Affiliations:** 1 Duke University Medical Center, Division of Hematopathology and Department of Pathology, Durham, NC USA; 2 Department of Hematopathology, The University of Texas MD Anderson Cancer Center, Houston, TX USA; 3 Department of Histology and Embryology, School of Basic Medical Science, Central South University, Changsha, Hunan China; 4 Department of Lymphoma and Myeloma, The University of Texas MD Anderson Cancer Center, Houston, TX USA; 5 Department of Lymphoma and Hematology, the Affiliated Tumor Hospital of Xiangya Medical School of Central South University, Changsha, Hunan China; 6 Department of Bioinformatics and Computational Biology, The University of Texas MD Anderson Cancer Center, Houston, TX USA; 7 Department of Medicine, Baylor College of Medicine, Houston, TX USA; 8 Servicio de Anatomía Patológica, Translational Hematopathology Lab, Hospital Universitario Marqués de Valdecilla/IDIVAL, Santander, Spain; 9 Fundacion Jimenez Diaz; CIBERONC, Madrid, Spain; 10 Duke Cancer Institute, Durham, NC USA

**Keywords:** Prognosis, Tumour immunology

## Abstract

Primary mediastinal large B-cell lymphoma (PMBCL) is a rare and distinct subtype of diffuse large B-cell lymphoma (DLBCL) without prognostic factors or a single standard of treatment clearly defined. In this study we performed retrospective analysis for clinical outcomes of 166 patients with PMBCL. In overall PMBCL, higher International Prognostic Index, stage, Ki-67 proliferation index, and positron emission tomography (PET) maximum standardized uptake values (SUVmax) at diagnosis were significantly associated with poorer survival, whereas MUM1 expression and higher peripheral blood lymphocyte/monocyte ratios were significantly associated with better survival. Patients who received R-HCVAD or R-EPOCH had better clinical outcome than did those who received the standard treatment R-CHOP. Treatment response and end-of-treatment PET SUVmax had remarkable correlations with survival outcome. In patients with refractory or relapsed PMBCL, stem cell transplant significantly improved overall survival. PMBCL had distinct gene expression signatures compared with overall DLBCL–NOS but not with DLBCL with *PD-L1*/*PD-L2* amplification. PMBCL also showed higher PD-L2 expression in B-cells, lower PD-1 expression in T-cells, and higher CTLA-4 expression in T-cells and distinct miRNA signatures compared with DLBCL-NOS. The prognostic factors, effectiveness of treatment, transcriptional and epigenetic signatures, and immunologic features revealed by this study enrich our understanding of PMBCL biology and support future treatment strategy.

## Introduction

Primary mediastinal large B-cell lymphoma (PMBCL) is a rare subtype of diffuse large B-cell lymphoma (DLBCL) with unique clinicopathologic features. PMBCL accounts for 2–4% of non-Hodgkin lymphomas and occurs most often in young females^1–3^. Clinically PMBCL often presents as a bulky mediastinal mass, commonly with local infiltration to the lung, chest wall, pleura, or pericardium. The World Health Organization has classified PMBCL as a unique entity^4^ on the basis of its unique clinical and immunophenotypic presentation and molecular features. PMBCL has a gene expression profile distinct from those of the germinal center B-cell (GCB) and activated B-cell (ABC) subtypes of DLBCL not otherwise specified (NOS) whereas shows features of nodular sclerosing Hodgkin lymphoma (a subtype of classic HL)^5,6^. Genetically, PMBCL has frequent copy number gains^5,7,8^ and translocation^9^ of the 9p24.1 locus resulting in *PD-L1*/*PD-L2/JAK2* overexpression, genetic alterations and downregulation of *CIIA* and MHC-II^10,11^ suggesting immune evasion, and a mutational profile suggesting relatedness to classic HL^12,13^. Recurrent mutations in PMBCL often affect the JAK/STAT and NF-κB pathways, in line with their constitutive activation^14–17^.

Given the rarity of PMBCL and lack of long-term follow-up data from large studies, there is no consensus on upfront treatment for PMBCL^1,14,18^. The role of consolidation radiation therapy in young PMBCL patients, who are predominantly female, also remains controversial because of long-term toxicity^2,19,20^. Before the rituximab era, dose-dense and dose-intense second- and third-generation protocols including MACOPB (methotrexate, leucovarin, doxorubicin, cyclophosphamide, vincristine, prednisone, and bleomycin) and VACOP-B (etoposide, doxorubicin, cyclophosphamide, vincristine, prednisone, and bleomycin) showed better clinical outcomes in PMBCL compared with the mainstay of treatment for DLBCL, anthracycline-containing regimen CHOP (cyclophosphamide, doxorubicin, vincristine, and prednisone)^20,21^. However, the addition of rituximab (R) to CHOP eliminated the difference^21–25^. In recent years, a single-arm clinical trial and retrospective studies have shown excellent clinical outcomes of PMBCL using the dose-adjusted (DA) EPOCH-R-regimen (etoposide, prednisone, vincristine, cyclophosphamide, doxorubicin, and rituximab) sparing patients from radiation therapy^26,27^. In the largest retrospective multicenter study of 132 PMBCL patients, compared with R-CHOP (*n* = 56), DA-EPOCH-R (*n* = 76) yielded higher complete response (CR) rates than R-CHOP (84% and 70%, respectively)^3^. However, in a recent retrospective study, R-CHOP-21 and DA-EPOCH-R had similar objective response rates (ORR) in 53 patients^28^. In both studies, DA-EPOCH-R did not improve overall survival (OS) or progression-free survival (PFS)^3,28^. Also, DA-R-EPOCH was associated with higher rates of neutropenic fever, infection complications, and hospitalization for acute toxicities^3^, leaving no consensus on the upfront treatment for PMBCL.

Furthermore, optimal salvage treatments for refractory or relapsed PMBCL are undergoing evolution. Considering the generally favorable clinical features (such as younger age and limited stage) of PMBCL patients, compared with typical DLBCL–NOS, PMBCL has a higher proportion of patients with primary refractory disease^3,24,29–31^, and refractory/relapsed PMBCL patients respond poorly to salvage therapies and have poor clinical outcome^31–33^. The standard treatment for relapsed/refractory DLBCL–NOS, high-dose chemotherapy followed by stem cell transplant (SCT) has shown clinical benefit, and response to induction chemotherapy was associated with better clinical outcome^28,29,34–36^. Recently, pembrolizumab, an anti-PD-1 immune checkpoint inhibitor, has been approved by FDA in PMBCL patients who have progressed after two or more lines of therapy (ORR > 40%), rendering a new treatment option for relapsed/refractory PMBCL especially for patients with high PD-L1 expression^37–39^. Another anti-PD-1 antibody nivolumab in combination with brentuximab vedotin (BV, an anti-CD30 antibody–drug conjugate) showed an even higher ORR of 73% in relapsed/refractory PMBCL^40^, although BV alone only showed an ORR of 13.3% in relapsed PMBCL^41^. However, grades 3 or 4 adverse events occurred in 53% of patients, compared with the 23–24% with pembrolizumab monotherapy. Moreover, anti-CD19 chimeric antigen receptor T (CAR-T) cell therapy with YESCARTA (axicabtagene ciloleucel) demonstrated a ORR of 82% and CR rate of 58% in a large cohort of refractory large B-cell lymphoma including eight cases of PMBCL^42^, and has been approved for the treatment of refractory DLBCL and PMBCL^38,43^. However, CAR-T cell therapy was associated with cytokine release syndrome, and neurological events and treatment-related death could occur.

Taking together, existing data regarding to a single standard of care are conflicting in PMBCL, and further exploration of genetic and epigenetic alterations in PMBCL is required to address unmet clinical needs. We thus sought to analyze whether different upfront, consolidation, and salvage therapies and clinicopathologic factors were associated with variable clinical outcome in a large cohort of PMBCL. Furthermore, we sought to delineate differences between PMBCL and DLBCL–NOS in epigenetic and immunobiologic expression by microRNA (miRNA) profiling and fluorescent multiplex immunohistochemistry, respectively.

## Subjects and methods

### Patients

We performed a retrospective analysis of 166 patients with PMBCL diagnosed during 2002–2014 at the Hospital Universitario Marques de Valdecilla/IDIVAL, Hunan Cancer Hospital, Duke University Medical Center, Baylor College of Medicine, Guangzhou Medical University and The University of Texas MD Anderson Cancer Center. Organized clinical data included age, sex, stage, B-symptoms, serum lactate dehydrogenase (LDH) level, mediastinal mass size, International Prognostic Index (IPI), Ann Arbor stage, extranodal sites, Eastern Cooperative Oncology Group (ECOG) performance status score, complete blood count (CBC), treatment (use of first-line regimen, radiation therapy, and SCT), response, and follow-up dates. Response was assessed by clinical examination and computed tomography scan or fluorodeoxyglucose positron emission tomography (PET)/computed tomography (CT) imaging of nodal regions according to the International Workshop to Standardize Response Criteria^44^. Imunohistochemical staining was performed to evaluate Ki-67 (MIB-1) proliferation index, CD30, CD5, CD23, CD10, MUM1, BCL2, and BCL-6 expression at the pathology labs, and data were available for collection in 89, 136, 81, 46, 114, 52, 68, and 77 patients, respectively. This study was reviewed and approved by the institutional review board and all patients have given informed consent in each of the collaboration centers.

### miRNA profiling and gene expression profiling

miRNA extracted from formalin-fixed, paraffin-embedded (FFPE) tissue samples was analyzed using the HTG EdgeSeq miRNA whole-transcriptome assay (HTG Molecular Diagnostics, Tucson, AZ), in which an excess of nuclease protection probes complementary to each miRNA hybridize to their targets. S1 nuclease then removes unhybridized probes and RNA, leaving behind only nuclease protection probes hybridized to their targets in a 1:1 ratio. Samples were individually barcoded (using a 16-cycle polymerase chain reaction to add adapters and molecular barcodes), individually purified using AMPure XP beads (Beckman Coulter), and quantitated using a KAPA Library Quantification Kit (Kapa Biosystems). Libraries were sequenced on the Illumina HiSeq platform (Illumina) for quantification. Standardization and normalization were provided to the project statistical core for validation of two pre-existing signatures and generation of new models (MCP clustering).

RNA extracted from 15 FFPE samples of PMBCL was transcribed into cDNA and applied to HG-U133 Plus 2.0 GeneChips (Affymetrix, Santa Clara, CA, USA). The profiling data were compared with those for EBV-negative DLBCL deposit in Gene Expression Omnibus GSE#31312 to identify differentially expressed genes with a false discovery rate (FDR) threshold using the beta-uniform mixture method^45^.

### Multiplex immunohistochemistry for immune checkpoint inhibitors

Multiplex immunohistochemistry was performed on 12 PMBCL patients using a series of antibodies with fluorescent conjugates and a MultiOmyx platform as did for DLBCL–NOS with methods described in details previously^46^, allowing detection and quantitative analysis of cell-specific expression of immune checkpoint molecules. Copy number alterations of *PD-L1*/*PD-L2* were examined using the fluorescent in situ hybridization (FISH) methods as previously described^46^.

### Statistical analysis

Overall survival (OS) was defined from the date of diagnosis to the last follow-up or death for any cause. Progression-free survival (PFS) was measured from the date of diagnosis to progression of disease or death from any cause. PFS and OS rates were compared by the Kaplan–Meier method. The associations between dichotomized factors and OS/PFS were analyzed using Log-rank (Mantel-Cox) test in the univariate analysis and Cox proportional hazard models in the multivariate analysis. Patient characteristics and response rates were compared using the Fisher’s exact test. Expression of immune markers was compared between two groups using unpaired Mann–Whitney test or Wilcoxon rank-sum test (two-tailed). Scattered plot was used to visualize the data points, mean, and the standard error of the mean in each group. In case of unequal variance of two groups by F-test of equality of variances (PD-L2 expression in this study), unpaired Welch *t*-test was used. *P*-values ≤ 0.05 were considered statistically significant. Statistical analyses were performed using GraphPad Prism 8.2.1 and IBM SPSS Statistics version 22.0.

## Results

### Patient characteristics

Supplementary Table 1 summarizes the clinical characteristics of the 166 PMBCL patients at diagnosis. Ninety-four patients were female, and 72 were male. The median age was 33 years (range, 11–64 years). One hundred twenty-one patients (73.8%) presented with stage I–II disease, and 117 patients (74.1%) were at low risk with a 0–1 IPI score. Sixty-five patients (39.4%) had B-symptoms, 106 (70.2%) patients had elevated serum LDH levels, 25 patients (15.3%) had an ECOG performance score of ≥2, 78 patients (52.7%) had a mediastinal mass size of >10 cm, and 28 patients (17.1%) had extranodal involvement. Initial staging PET/CT showed maximum standardized uptake values (SUV_max_) of >11.6 in 82 patients (77.4%). CD30 was positive in 72.1% of patients.

### Prognostic factors at diagnosis

Clinicopathologic variables were analyzed for associations with treatment response and survival outcomes. Patients who achieved CR with front-line treatment compared with those who did not had significantly lower frequency of B-symptoms (32% vs 56%, *P* = 0.0053) and higher frequency of BCL-6 expression (95% vs 74%, *P* = 0.019) (Supplementary Table 2).

**Fig. 1:**
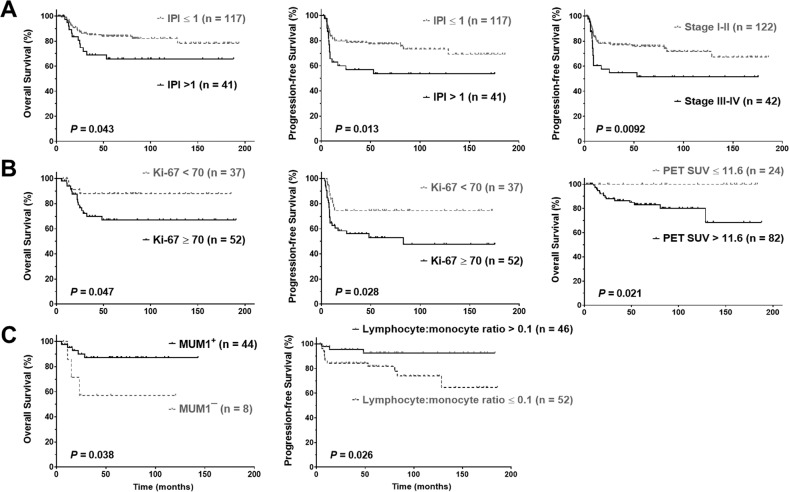
Prognostic factors at diagnosis in PMBCL. **a** IPI > 1 was associated with poorer overall and progression-free survival; advanced stage was associated with poorer progression-free survival. **b** Ki-67 ≥ 70% was associated with poorer overall and progression-free survival; PET SUV_max_ > 11.6 (≥12.8 in this cohort) was associated with poorer overall survival. **c** MUM1 positivity and higher lymphocyte:monocyte ratio were associated with better overall and progression-free survival, respectively. *PMBCL* primary mediastinal large B-cell lymphoma, *IPI* International Prognostic Index, *PET* positron emission tomography, *SUV*_*max*_ maximum standardized uptake value.

With a median follow-up of 58 months (range, 3–194 months), the overall 5-year OS and PFS rates were 79% and 70%, respectively. An IPI of >1 and Ki-67 expression of ≥70% were found to be associated with significantly poorer OS and PFS (Fig. 1a, b). Patients with stage III–IV disease had significantly poorer PFS (*P* = 0.0092, Fig. 1a; *P* = 0.065 for OS, Supplementary Fig. 1). Elderly patients (age > 60, only three patients) and those with PET SUV_max_ of >11.6 at diagnosis had significantly poorer OS (*P* = 0.023, Supplementary Fig. 1, and *P* = 0.021, respectively). In contrast, MUM1/IFR4 positivity and higher CBC lymphocyte/monocyte ratio at diagnosis were associated with significantly better OS (*P* = 0.038) and PFS (*P* = 0.026), respectively (Fig. 1c).

In contrast, sex, ECOG performance status, size of the mediastinal mass, B-symptoms, pleural effusion, involvement in the superior vena cava, cerebrospinal fluid, or bone marrow, and other clinical features did not show a significant prognostic impact. Elevated serum LDH level only showed a slight trend toward unfavorable OS, whereas CD30 positivity and high absolute lymphocyte counts were associated with a trend of better PFS with a marginal *P*-value (*P* = 0.066 and 0.072, respectively, Supplementary Fig. 1).

In multivariable survival analysis for factors that were significant on univariate analysis, the unfavorable prognostic effect of Ki-67 on OS (*P* = 0.023, hazard ratio [HR] = 10.64, 95% CI 1.38–81.80) and PFS (*P* = 0.006, HR = 4.54, 95% CI 1.54–13.40), the favorable prognostic effects of MUM1 positivity on OS (*P* = 0.045, HR = 0.12, 95% CI 0.015–0.96), and the favorable prognostic effects of high CBC lymphocyte/monocyte ratio on PFS (*P* = 0.048, HR = 0.24, 95% CI 0.06–0.99) remained significant after adjustment for clinical parameters (Supplementary Table 3). However, high PET SUV_max_ at diagnosis, CD30 positivity, and absolute lymphocyte counts were not significant factors in the multivariate analysis.

### Prognostic factors at the end of treatment

**Fig. 2:**
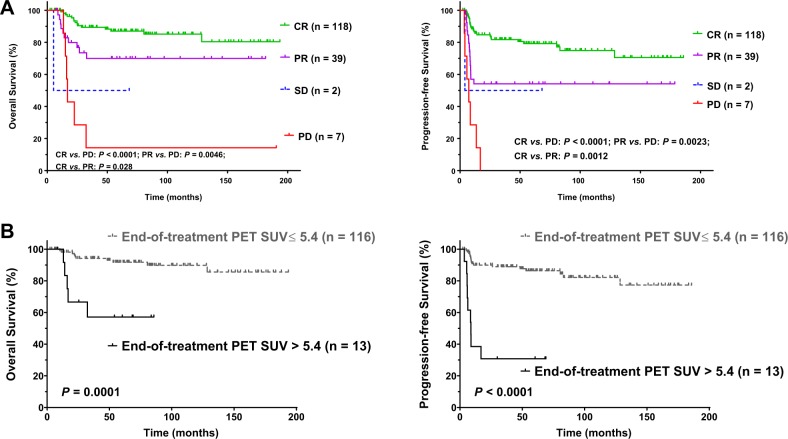
Prognostic factors after treatment in PMBCL. Treatment response (**a**) and end-of treatment PET SUV_max_ (**b**) were associated with overall and progression-free survival. *PMBCL* primary mediastinal large B-cell lymphoma, *CR* complete response, *PR* partial response, *SD* stable disease, *PD* progressive disease, *PET* positron emission tomography, *SUV*_*max*_ maximum standardized uptake value.

After completion of the scheduled treatment, 118 patients had a CR (71.1%), 39 patients had a partial response (23.5%) (yielding an ORR of 94.6%), two patients (1.2%) had stable disease, and seven patients (4.2%) had progression of disease. Upon PET/CT at the end of treatment, 116 patients (89.9%) obtained a SUV_max_ of ≤5.4, and these patients did not show significant difference in clinical features at diagnosis compared with patients with a SUV_max_ of >5.4 (Supplementary Table 4). Both treatment response (Fig. 2a) and end-of-treatment PET SUV_max_ (Fig. 2b) were remarkably significantly associated with OS and PFS. In the multivariate analysis adjusting for clinical variables, the unfavorable prognostic significance of end-of-treatment PET SUV_max_ was remarkable on PFS (*P* < 0.001, HR = 6.21, 95% CI 2.24–17.17) and marginal on OS (*P* = 0.071).

### Differential efficacy of upfront and salvage treatments

**Fig. 3:**
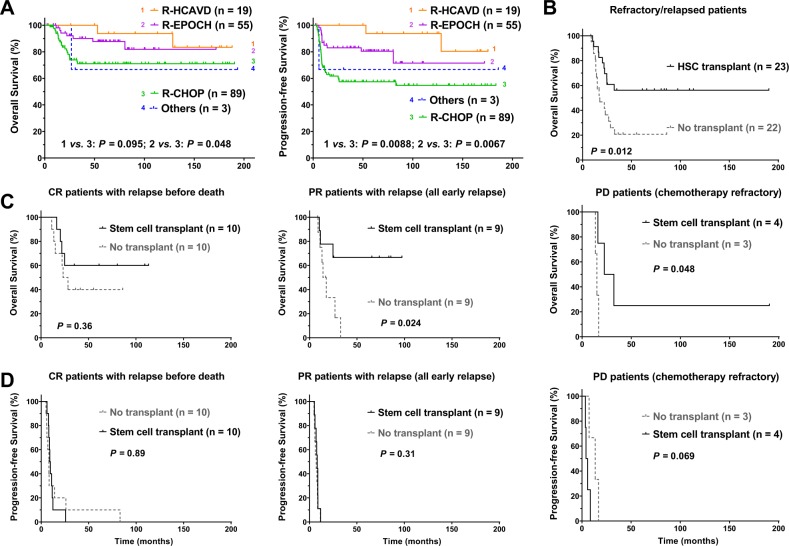
Treatment options and prognosis in PMBCL. **a** R-HCVAD and R-EPOCH appeared to be more effective than R-CHOP. **b** Stem cell transplant (autologous in most cases) improved overall survival in patients with relapsed/refractory PMBCL. **c** Among relapsed/refractory cases, relapsed patients who had partial-response to frontline therapy and refractory patients with progressive disease benefited the most from stem cell transplant. **c** Relapsed/refractory PMBCL patients with and without stem cell transplant had simiarly poor progression-free survival. *PMBCL* primary mediastinal large B-cell lymphoma, *R-HCVAD* rituximab, cyclophosphamide, mesna, doxorubicin, vincristine, dexamethasone, methotrexate, and cytarabine, *R-EPOCH* rituximab, etoposide, prednisone, vincristine, cyclophosphamide, and doxorubicin, *R-CHOP* rituximab, cyclophosphamide, doxorubicin, vincristine, and prednisone, *CR* complete response, *PR* partial response, *PD* progressive disease.

Grouping the study cohort by the primary treatments, most (*n* = 89) patients were treated with R-CHOP alone at the front-line, whereas 55 patients were treated with 4–7 cycles (mostly six cycles) of R-EPOCH (five people also received prior 1–3 cycles of R-CHOP), 19 patients were treated with 3–8 cycles (mostly six cycles) of R-HCVAD (rituximab, cyclophosphamide, mesna, doxorubicin, vincristine, dexamethasone, methotrexate, and cytarabine; four people also received prior 1–3 cycles of R-CHOP), and the other three patients were treated with other regimens. Compared with the R-CHOP-alone group, the R-HCVAD group had significantly higher percentage of patients with a high IPI score, elevated serum LDH, a high ECOG performance status, and a low CBC lymphocyte/monocyte ratio. The R-EPOCH group had significantly higher percentage of patients with high PET SUV_max_ at diagnosis and nonsignificant trends towards high ECOG and IPI (Table 1). Regarding to clinical outcome, R-HCVAD and R-EPOCH regimens compared with the standard R-CHOP treatment were associated with higher ORR (100%, 98%, and 91%, respectively), but the difference was not statistically significant. However, with a median follow-up duration of 56.8 month, 126.2 month, and 54.8 month for R-EPOCH, R-HCVAD, and R-CHOP, respectively, we found that patients treated with R-EPOCH or R-HCVAD had significantly better OS and/or PFS than did patients treated with R-CHOP (Fig. 3a).

**Table 1:** Clinicopathologic characteristics of patients with PMBCL treated with different chemotherapy regimens.

First-line treatment	R-CHOP	R-EPOCH	R-HCAVD		
Characteristic	*n* (%)	*n* (%)	*P*1	*n* (%)	*P*2
Age					
≤60 years	89 (100)	53 (96.4)	0.14	18 (94.7)	0.18
>60 years	0 (0)	2 (3.6)		1 (5.3)	
Sex					
Female	51 (57.3)	29 (52.7)	0.61	13 (68.4)	0.45
Male	38 (42.7)	26 (47.3)		6 (31.6)	
Stage					
I–II	67 (77.0)	39 (70.9)	0.43	12 (63.2)	0.25
III–IV	20 (23.0)	16 (29.1)		7 (36.8)	
B-symptoms					
No	53 (59.6)	33 (61.1)	1.0	12 (63.2)	1.0
Yes	36 (40.4)	21 (38.9)		7 (36.8)	
Serum LDH					
Normal	29 (37.2)	13 (25)	0.18	1 (5.6)	**0.0098**
Elevated	49 (62.8)	39 (75)		17 (94.4)	
No. of extranodal involvement					
0–1	84 (96.6)	52 (94.5)	0.68	16 (84.2)	0.069
≥2	3 (3.4)	3 (5.5)		3 (15.8)	
ECOG performance status					
0–1	81 (92.0)	43 (81.1)	0.065	12 (63.2)	**0.003**
≥2	7 (8.0)	10 (18.9)		7 (36.8)	
Mediastinal mass size					
≤10 cm	40 (54.8)	22 (41.5)	0.15	7 (36.8)	0.20
>10 cm	33 (45.2)	31 (58.5)		12 (63.2)	
IPI risk group					
0–1	67 (81.7)	37 (68.5)	0.098	11 (57.9)	**0.035**
>1	15 (18.3)	17 (31.5)		8 (42.1)	
Treatment response					
Complete	57 (64.0)	44 (80.0)	0.06^a^	16 (84.2)	0.11^a^
Partial	24 (27.0)	10 (18.2)		3 (15.8)	
Stable disease	2 (2.2)	0 (0)		0 (0)	
Progressive disease	6 (6.7)	1 (1.8)		0 (0)	
Ki-67 index					
<70%	17 (37.8)	18 (51.4)	0.26	2 (25)	0.70
≥70%	28 (62.2)	17 (48.6)		6 (75)	
PET SUV_max_ at diagnosis					
≤11.6	16 (31.4)	4 (10.5)	**0.023**	4 (25)	0.76
>11.6	35 (68.6)	34 (89.5)		12 (75)	
End-of-treatment PET SUV_max_					
≤5.4	53 (86.9)	44 (89.8)	0.77	17 (100)	0.19
>5.4	8 (13.1)	5 (10.2)		0 (0)	
CBC lymphocyte count					
≤1.2.1 × 10^9^/L	22 (64.7)	25 (58.1)	0.64	14 (73.7)	0.56
>1.2.1 × 10^9^/L	12 (35.3)	18 (41.9)		5 (26.3)	
CBC lymphocyte:monocyte ratio					
≤0.1	14 (41.2)	22 (51.2)	0.49	14 (73.7)	**0.043**
>0.1	20 (58.8)	21 (48.8)		5 (26.3)	
CD30 expression					
Negative	20 (27.8)	15 (31.3)	0.69	2 (13.3)	0.34
Positive	52 (72.2)	33 (68.8)		13 (86.7)	
MUM1 expression					
Negative	4 (22.2)	4 (13.8)	0.69	0 (0)	0.55
Positive	14 (77.8)	25 (86.2)		4 (100)	
Radiation therapy					
No	10 (15.6)	8 (30.8)	0.14	1 (7.7)	0.68
Yes	54 (84.4)	18 (69.2)		12 (92.3)	
Stem cell transplant					
No	70 (78.7)	46 (83.6)	0.52	17 (89.5)	0.36
Yes	19 (21.3)	9 (16.4)		2 (10.5)	

Significant *P*-values are in bold.

*PMBCL* primary mediastinal large B-cell lymphoma, *LDH* lactate dehydrogenase, *ECOG* Eastern Cooperative Oncology Group, *IPI* International Prognostic Index, *R-CHOP* rituximab, cyclophosphamide, doxorubicin, vincristine, and prednisone, *R-EPOCH* rituximab, etoposide, prednisone, vincristine, cyclophosphamide, and doxorubicin, *R-HCVAD* rituximab, cyclophosphamide, mesna, doxorubicin, vincristine, dexamethasone, methotrexate, and cytarabine, *PET* positron emission tomography, *SUV*_*max*_ maximum standardized uptake value, *CBC* complete blood count.

*P1* R-EPOCH vs R-CHOP group, *P2* R-HCVAD vs R-CHOP group.

^a^CR vs. non-CR.

Radiation therapy was administered in 85 of 106 patients (80.2%, all except three patients achieved CR/partial response prior to radiotherapy) at the discretion of the treating physician. Radiation therapy did not show clinical benefit in the overall cohort (Supplementary Fig. 2A) or subgroups of patients categorized according to treatment regimens or treatment response. In the relapsed/refractory setting, radiotherapy was administrated in only six patients and did not show impact on survival in relapsed/refractory PMBCL (Supplementary Fig. 2B).

Totally 31 patients of the cohort (18.7%) received SCT, including 25 cases auto-SCT and six cases allo-SCT. Among these 31 patients, 11 patients had CR to the first-line treatment, 16 patients had partial response, and four patients had progressive disease after the first-line treatment (Supplementary Table 4). Nineteen of the 27 patients with CR or partial responsive disease had a relapse after treated with R-CHOP or R-EPOCH. These 19 relapsed patients plus four patients with primary resistant PMBCL who underwent SCT had significantly better clinical outcome compared with 22 patients with relapsed/refractory PMBCL without receiving SCT treatment (*P* = 0.012, Fig. 3b). Among patients undergoing SCT, time to relapse from diagnosis did not show correlation with OS, although only two patients had relapsed ≥1 year and had a trend of better survival (*P* = 0.23). The salvage chemotherapy in relapsed/refractory PMBCL patients, including RICE (rituximab, ifosfamide, carboplatin, and etoposide), HCVAD, RDHAP, ESHAP, and other chemotherapies or BV, were not significantly different between the SCT and non-SCT group. Notably, in the non-SCT group, one relapsed PMBCL patient was treated with combined velcade (bortezomib) and rituximab treatment after two cycles of RICE and expired at 11 month follow-up; and one relapsed patient received RICE, RDHAP, and anti-CD19 CAR-T cell therapy who died in the 23rd month from diagnosis (8 month from relapse).

To examine whether the efficacy of SCT was limited in certain patient groups, we divided patients according to upfront chemotherapy and treatment response. The clinical benefit of SCT in relapsed/refractory PMBCL was independent of first-line chemotherapy (Supplementary Fig. 3A). However, the favorable effect of SCT on OS was not significant in CR patients who experienced a relapse after first-line treatment, but was significant in relapsed patients after a partial response to first-line treatment (*P* = 0.024) and in patients with progressive disease (refractory; *P* = 0.048), even though two patients received allo- but not auto-SCT (Fig. 3c). The duration of relapse time from diagnosis did not significantly differ between patients who received SCT and those who did not receive SCT, although there was a trend towards shorter PFS duration for PD patients who received salvage SCT (Fig. 3d). For PMBCL patients without relapsed/refractory disease, the clinical outcomes with and without SCT were all excellent (unreached OS) except one CR patient (died at 53 month from diagnosis) not allowing survival comparison (Supplementary Fig. 3B).

### Immune checkpoint expression in PMBCL versus DLBCL–NOS

**Fig. 4:**
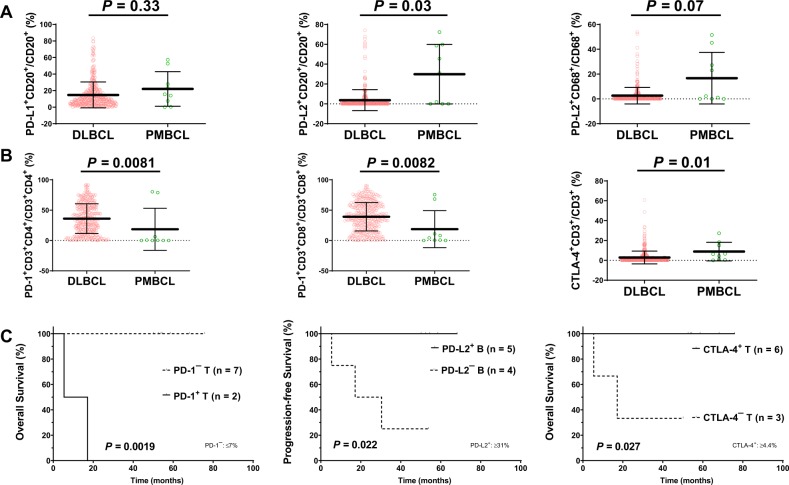
Immune checkpoint expression in PMBCL versus DLBCL. **a** Compared with de novo DLBCL, PMBCL expressed significantly higher levels of PD-L2 in B-cells. **b** Compared with de novo DLBCL, PMBCL expressed significantly lower levels of PD-1 in T-cells but higher levels of CTLA-4 in T-cells. **c** Prognostic significance of PD-1 expression in T-cells, CTLA-4 expression in T-cells, and PD-L2 in B-cells in PMBCL. *PMBCL* primary mediastinal large B-cell lymphoma, *DLBCL* diffuse large B-cell lymphoma.

As immunotherapies have become new standard of care options in relapsed/refractory PMBCL, to gain further insight into the tumor microenvironment of PMBCL, we used fluorescence multiplex immunohistochemistry technology to analyze the expression of immune checkpoint molecules on both the tumor and immune cells in nine samples of PMBCL, and compared the expression results with those of de novo DLBCL–NOS^46^. Compared with DLBCL–NOS, PMBCL did not show significantly higher PD-L1 expression in B-cells, but showed significantly higher levels of PD-L2 expression in B-cells (*P* = 0.03; Fig. 4a), lower PD-1 expression in CD8 or CD4-positive T-cells (*P* = 0.0081 and 0.0082, respectively), and higher CTLA-4 expression (*P* = 0.01) in T-cells (Fig. 4b). FISH was performed in 10 PMBCL cases and identified one case with *PD-L1*/*PD-L2* amplification, one case with *PD-L1*/*PD-L2* copy number gain, one case with polyploidy, and one case with *PD-L2* (but not *PD-L1*) amplification (total alteration rate: 40%). *PD-L2* (but not *PD-L1*) copy number alteration showed a nonsignificant trend towards higher PD-L2 expression (*P* = 0.14). On the other hand, the percentage of T-cells (CD4 or CD8) or macrophages in the tumor microenvironment was similar between PMBCL and DLBCL–NOS. PMBCL had a slight trend of higher tumor-infiltrating natural killer cells compared with DLBCL–NOS but the difference was not significant (*P* = 0.17).

We further correlated the expression levels of PD-1, PD-L1, PD-L2, and CTLA-4 with clinical outcome in PMBCL. PD-1 expression in T-cells was associated with significantly poorer OS and PFS, whereas CTLA-4 expression in T-cells and PD-L2 expression in B-cells were associated with significantly better OS and PFS, respectively (Fig. 4c, Supplementary Fig. 4), resembling the prognostic impact of immune checkpoint expression in DLBCL–NOS^46^. *PD-L1*/*PD-L2* copy number alteration was associated with trend of better PFS but the *P-*value was not significant (OS, *P* = 0.23; PFS, *P* = 0.098).

### Gene expression profiling in PMBCL versus DLBCL

Gene expression profiling was performed in 15 PMBCL cases and the profiles were compared with those of de novo DLBCL–NOS^45^. PMBCL showed significantly differentially expressed genes compared with all the DLBCL molecular subtypes (GCB–DLBCL, ABC–DLBCL, and unclassifiable DLBCL; Table 2, Supplementary Fig. 5), including those in previously identified PMBCL molecular signatures^5,6,47^, such as upregulation of *CCL17*, *IL13RA1*, *MST1R*, *MOBKL2C*, *SLAMF1*, *TRAF1*, *TFPI2*, and *TMOD1* and downregulation of *FOXP1*, *IGHM*, *IKZF1*, *PIM2*, and *TNFRSF13B*, which suggest activation of the JAK/STAT and NF-κB pathways and T-cells/macrophages and downregulation of the B-cell receptor pathway. Other notable PMBCL signatures include upregulation of *CCL22* and downregulation of *GNA13* compared with GCB–DLBCL; upregulation of *ICOSLG* (inducible T cell costimulator ligand), *C17orf99, AKT1S1*, and *KDM4B* and downregulation of *MPEG1* (macrophage expressed 1), *LYN* (involved in the B-cell receptor signaling regulation), *MAP3K1*, and *UBA5* compared with ABC–DLBCL; and upregulation of *ASB12* and *MORC2* and downregulation of *IGK@/IGKC* compared with unclassifiable DLBCL.

**Table 2:** Significantly differentially expressed genes between PMBCL and DLBCL subtypes determined by gene expression profiling analysis.

Comparison	Upregulated genes	Downregulated genes
PMBCL vs. GCB–DLBCL (FDR = 0.01)	CCL17*, *CCL22*, *MYH6*, *KCNT2*, *HP*, *VASH2*, *C11orf9*, *BCHE*, *CYP21A2*, *NCRNA00184*, *MYO5C*, *PLLP*, *KCTD14*, MST1R*, *ALDH1B1*, *HOXB3*	*SMEK1*, *GNA13*
PMBCL vs. ABC–DLBCL (FDR = 0.01, fold-change > 1.5)	*C17orf99*, CCL17*, IL13RA1*, TRAF1*, *CACNA1E*, ***TFPI2***, *STAG3*, *KIAA1466*, *AUTS2*, *AKT1S1*, *CCDC80*, *ATP8A1*, *SNX29*, *NEK6*, *ABCC4*, *TBX5*, *hCG_1983896*, *CUX2*, *FAM171B*, ***TMOD1***, *PTGIS*, *RBM9*, *BCHE*, *LOC553137*, SLAMF1*, *LOC100129034*, *KDM4B*, *SYTL4*, *TCTN1*, ***MOBKL2C***, *NCRNA00184*, *ICOSLG*	*IGHM*, *P2RX5*, *TNFRSF13B*, *FUT8*, *MPEG1*, *FOXP1*, *SLC38A1*, *LYN*, *IKZF1*, *MAP3K1*, *BZW2*, ***PIM2***, *UBA5*
PMBCL vs. unclassifiable DLBCL (FDR = 0.2)	*NID1*, *TAL1*, *ASB12*, *GYPA*/*GYPB*, *GABRA2*, *ABHD1*, *KRR1*, *RAPGEF1*, *MORC2*, *KCNK9*, *AGPAT3*, *PUSL1*, *SFT2D1*, *RTN4IP1*	*IGK@*/*IGKC*, *ZNF514*

Shared gene signatures with Mottok et al. 2019 (ref. ^11^) were in bold; shared gene signatures with Savage et al. 2003 (ref. ^6^) were underlined; shared gene signatures with Rosenwald et al. 2003 (ref. ^5^) were marked by asterisk (*).

*PMBCL* primary mediastinal large B-cell lymphoma, *DLBCL* diffuse large B-cell lymphoma, *GCB* germinal center B-cell DLBCL, *ABC* activated B-cell DLBCL, *FDR* false discovery rate.

As a most recent study found that the gene expression profile of DLBCL with 9p24 amplification was similar to those of PMBCL^48^, we compared the 15 PMBCL cases with 15 DLBCL–NOS cases with *PD-L1*/*PD-L2* amplifications (almost exclusively ABC-subtype)^46^. No significantly differentially expressed genes were identified between PMBCL and DLBCL–NOS with *PD-L1*/*PD-L2* amplifications.

### miRNA expression profiles in PMBCL versus DLBCL

**Fig. 5:**
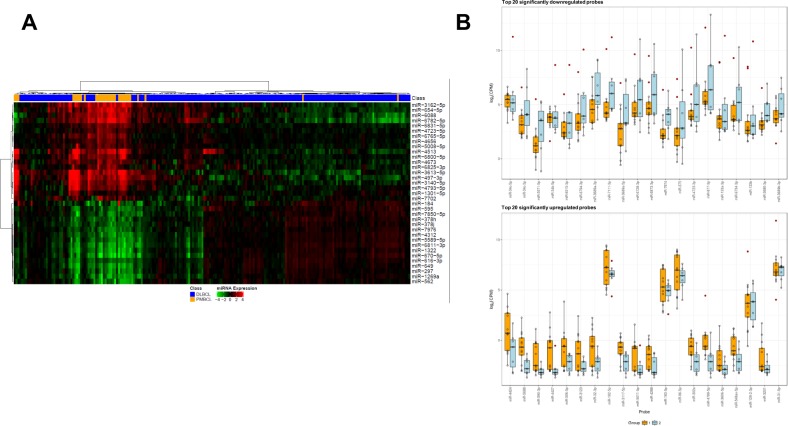
miRNA profiles in PMBCL versus DLBCL. **a** Heatmap for top 20 upregulated and 15 downregulated miRNAs in PMBCL compared with de novo DLBCL. **b** Scatter plots for top 20 upregulated and downregulated miRNAs in PMBCL patients with PFS events (blue bars) compared with those without events (brown bars). *PMBCL* primary mediastinal large B-cell lymphoma, *DLBCL* diffuse large B-cell lymphoma.

As miRNAs are important regulators of gene expression^49^ and B-cell development^50^, we performed miRNA profiling in 43 PMBCL cases and compared miRNA expression between PMBCL and de novo DLBCL–NOS cases^51–53^. A large number of miRNAs were significantly differentially expressed between de novo DLBCL–NOS and PBMCL with a FDR threshold of 0.05. The top 20 significant miRNAs with FDR < 0.01 were listed in Table 3 and the differential expression was visualized in Fig. 5a. Half of the miRNAs in Table 3 were functionally studied in different types of cancers but not in lymphomas (however, the oncogenic/tumor suppressor/drug resistance roles of these miRNAs were often inconsistent), although a previous study found miR-497 expression was associated with chemosensitivity and better survival in DLBCL^54^.

**Table 3:** Significantly differentially expressed miRNAs between PMBCL and de novo DLBCL–NOS (top 20 upregulated and 20 downregulated miRNAs) and between PMBCL patients with PFS events and those without PFS events by miRNA profiling analysis.

miRNA name	Differential expression		AveExpr	Fold-change 2 vs 1		Adjusted p 2 vs 1
	PMBCL vs DLBCL–NOS		All cases	(Log)	Raw p 2 vs 1	Benjamini–Hochberg (FDR 0.05)
miR-6800-5p	Up		9.78	**6.41**	0.00	0.00
miR-4513	Up		8.14	**8.34**	0.00	0.00
miR-4793-5p	Up		5.97	**4.70**	0.00	0.00
miR-7702	Up		6.59	**3.20**	0.00	0.00
miR-3140-5p	Up		5.47	**5.01**	0.00	0.00
miR-3162-5p	Up		8.08	**5.71**	0.00	0.00
miR-5008-5p	Up		5.69	**2.77**	0.00	0.00
miR-6825-3p	Up		9.57	**3.25**	0.00	0.00
miR-3613-5p	Up		3.97	**8.04**	0.00	0.00
miR-497-3p	Up		6.29	**6.45**	0.00	0.00
miR-6782-5p	Up		9.79	**6.14**	0.00	0.00
miR-6088	Up		13.79	**4.79**	0.00	0.00
miR-4673	Up		4.89	**3.31**	0.00	0.00
miR-184	Up		7.18	**4.49**	0.00	0.00
miR-6831-5p	Up		7.58	**2.61**	0.00	0.00
miR-6765-5p	Up		10.34	**2.71**	0.00	0.00
miR-654-5p	Up		9.38	**4.50**	0.00	0.00
miR-4723-5p	Up		6.23	**2.40**	0.00	0.00
miR-4656	Up		10.32	**2.30**	0.00	0.00
miR-1301-5p	Up		7.16	**2.31**	0.00	0.00
miR-1322	Down		11.50	−2.84	0.00	0.00
miR-649	Down		12.27	−2.60	0.00	0.00
miR-297	Down		9.34	−2.23	0.00	0.00
miR-1269a	Down		7.91	−2.33	0.00	0.00
miR-670-5p	Down		8.16	−2.90	0.00	0.00
miR-616-3p	Down		10.71	−2.10	0.00	0.00
miR-562	Down		5.88	−2.26	0.00	0.00
miR-378h	Down		8.15	−1.99	0.00	0.00
miR-595	Down		8.33	−2.20	0.00	0.00
miR-7976	Down		7.43	−1.87	0.00	0.00
miR-5589-5p	Down		9.76	−2.11	0.00	0.00
miR-4312	Down		9.14	−2.10	0.00	0.00
miR-378j	Down		7.49	−1.93	0.00	0.00
miR-7850-5p	Down		7.52	−2.01	0.00	0.00
miR-6811-3p	Down		9.93	−2.44	0.00	0.00
miR-548ay-5p	Down		11.59	−2.37	0.00	0.00
miR-4273	Down		6.63	−2.61	0.00	0.00
miR-5586-3p	Down		7.65	−1.74	0.00	0.00
miR-4483	Down		6.38	−2.28	0.00	0.00
miR-4502	Down		10.00	−1.71	0.00	0.00

	PMBCL, progression vs PMBCL, no progression					
miR-4424	Down		3.92	−10.76	0.00	0.23
miR-5688	Down		1.13	−9.73	0.00	0.23
miR-34c-5p	Up		9.69	**6.04**	0.00	0.24
miR-590-3p	Down		−0.17	−13.48	0.00	0.24
miR-34c-3p	Up		7.13	**3.84**	0.00	0.33
miR-4427	Down		1.10	−13.26	0.00	0.33

Fold change of upregulation was in bold.

To identify clinically relevant epigenetic signatures, we compared miRNA profiles of PMBCL patients with or without a progression/relapse event by *t*-test (Supplementary Fig. 6). Multiple miRNAs were differentially expressed between PMBCL patients with a PFS event and those without (Fig. 5b); however, the differences were not significant with the FDR threshold of 0.05 after multiple testing correction. The five miRNAs with the lowest FDR were listed in Table 3.

## Discussion

Understanding the unique pathobiology and treatment of PMBCL has paved the way for high chances of cure in patients^17,29,38^. To add informative data to PMBCL studies, we performed a multicenter retrospective study in a up-to-date largest cohort of 166 patients with PMBCL. The study is among the largest studies of prognostic factors and treatment in PMBCL, and is the first study of miRNA expression profiling in PMBCL. We found that IPI, stage, Ki-67 (consistent with previous studies^19,26,55^) MUM1 positivity (different from a previous study^56^), and CBC lymphocyte/monocyte ratio (a novel independent favorable prognostic factor identified in this study) significantly affected OS and PFS. Baseline PET SUV_max_ was significantly associated with inferior PFS, underscoring the predictive power of PET–CT imaging. After chemotherapy, the treatment response and end-of-treatment PET SUV_max_ were significantly associated with clinical outcome. The prognostic value of PET SUV_max_ is consistent with the results of the International Extranodal Lymphoma Study Group IELSG–26 trial, in which prospectively enrolled patients with a Deauville score of 1–3 and those with a score of >3 at the end of treatment had a 5-year PFS rate of 99% of 68%, respectively^57^, suggesting that end-of-therapy PET–CT imaging can serve as a disease assessment tool. However, these prognostic effects will be more valuable and clinically significant when they are validated by future independent studies.

Currently there is a lack of consensus on optimal chemo/radiotherapy for PMBCL. Existing retrospective results are conflicting with regard to a single standard of care^3,23,28,58^. Our data from a large cohort of PMBCL show that front-line R-EPOCH and R-HCVAD were both associated with superior long-term OS and/or PFS compared with R-CHOP, corroborating an earlier study that showed therapeutic advantage of R-EPOCH over R-CHOP with short follow-up^3^. However, we acknowledge the retrospective nature of this study with careful interpretation of the results. Radiotherapy was not associated with prognostic effects in our cohort either in the consolidation or salvage setting. Furthermore, our study demonstrated that SCT significantly improved the OS of patients with relapsed or refractory PMBCL, particularly for relapsed patients with an initial partial response and patients with progressive (refractory) disease. Among relapsed PMBCL without SCT, one patient received CAR-T cell therapy but had a shorter survival than most patients who underwent SCT. The estimated 3-year OS of relapsed/refractory PMBCL patients with and without SCT was 56.2% and 20.8%, respectively, comparable to the estimated 3-year OS of 61% for transplant-eligible relapsed/refractory patients treated with uniform salvage therapy with intent of subsequent high-dose therapy and autologous SCT^35^ and 5-year OS of 45%^36^ by recent studies. However, SCT as consolidation therapy^28^ did not show additional benefit in our cohort. These results provides further support that high-dose chemotherapy followed by SCT should be highly recommended for treatment of relapsed or refractory PMBCL^1,18^.

PD-1 and CTLA-4 are immune checkpoint receptors that transmit an inhibitory signal to T-cells after ligation and contribute to exhaustion of tumor-infiltrating T-cells. Immune checkpoint blockade therapy has demonstrated impressive efficacy in PMBCL and various advanced cancers. Although the ORR to anti-PD-1 monotherapy was lower in PMBCL than in classical HL, nivolumab and BV combination therapy has shown a high ORR of 73% in relapsed/refractory PMBCL without treatment-related death^40^. *PD-L1* genetic alterations and expression have been shown to be associated with the efficacy of anti-PD-1 therapy immunotherapy^1,14,18^. Therefore, we evaluated the tumor microenvironment of PMBCL and found that compared with DLBCL–NOS, PMBCL had significantly higher levels of PD-L2 expression in B-cells and higher CTLA-4 expression whereas lower PD-1 expression in T-cells. The higher expression of PD-L2 rather than PD-L1 was also reported by recent studies^59,60^. Our results suggest that both PD-1 and CTLA-4 inhibitors have therapeutic potential in PMBCL^14^; however, the case numbers were small and future studies are warranted to confirm these observations.

Molecular studies of PMBCL have shown that although PMBCL is categorized as a DLBCL subtype by morphology, PMBCL has a gene transcriptional signature distinct from DLBCL and more similar to that of nodular sclerosing Hodgkin lymphoma^1,2,18^. However, our data and a previous study^48^ found that the gene expression profiles of PMBCL were not significantly different from those of DLBCL–NOS with *PD-L1*/*PD-L2* amplification. Furthermore, in this study we compared miRNA profiles between DLBCL and PMBCL, and identified PMBCL miRNA signatures. As miRNAs are fine-tuners of gene expression and regulators of biochemical pathways including tumorigenesis pathways, these miRNAs may represent interesting therapeutic targets and novel biomarkers. The epigenetic features of PMBCL remain to be further explored. It will be interesting to further compare gene and miRNA expression profiles between PMBCL and classic HL with *PD-L1*/*PD-L2* amplification.

In summary, in this large multicentric cohort analysis, we demonstrated that R-HCVAD and R-EPOCH provide superior clinical outcomes in PMBCL than R-CHOP as front-line immunochemotherapy that should be integrated into the clinical practice guidelines. For relapsed or refractory PMBCL, autologous or allogenic SCT after high-dose chemotherapy as the standard of care is an effective therapeutic strategy for PMBCL. High expression of PD-L2 and CTLA-4 and low expression of PD-1 in the tumor microenvironment of PMBCL suggest efficacy of immune checkpoint blockade for SCT-ineligible patients or patients with refractory disease. The unique gene expression and miRNA signatures of PMBCL may represent important regulators and pathways of tumorigenesis and hence drug targets.

## Supplementary information

Supplemental Tables

Supplemental Figures
