# Supplementary material for: Prognostic factors, therapeutic approaches, and distinct immunobiologic features in patients with primary mediastinal large B-cell lymphoma on long-term follow-up
Source: Blood Cancer J. 2020 May 4;10(5):49. doi: 10.1038/s41408-020-0312-7 (PMC7198569; doi:10.1038/s41408-020-0312-7)
Supplement: Supplementary file 1 — Supplemental Tables [file 41408_2020_312_MOESM1_ESM.pdf]

Supplementary Table 1. Clinicopathologic characteristics of patients in the overall cohort

Characteristic	n (%)
**Age**	
≤60 years	163 (98.2)
>60 years	3 (1.8)
**Sex**	
Male	72 (43.4)
Female	94 (56.6)
**Stage**	
I-II	121 (73.8)
III-IV	43 (26.2)
**B symptoms**	
No	100 (60.6)
Yes	65 (39.4)
**Serum LDH**	
Normal	45 (29.8)
Elevated	106 (70.2)
**No. of extranodal sites**	
0-1	155 (94.5)
≥2	9 (5.5)
**ECOG performance status**	
0-1	138 (84.7)
≥2	25 (15.3)
**Mediastinal mass size**	
≤10 cm	70 (47.3)
>10 cm	78 (52.7)
**IPI risk group**	
0-1	117 (74.1)
>1	41 (25.9)
**First-line Chemotherapy**	
R-CHOP	89 (53.6)
R-EPOCH	55 (33.1)
R-HCVAD	19 (11.4)
Others	3 (1.8)
**Treatment response**	
Complete	118 (71.1)
Partial	39 (23.5)
Stable disease	2 (1.2)
Progressive disease	7 (4.2)
**Ki-67**	
<70%	37 (41.6)

≥70%	52 (58.4)
**PET SUV_{max} at diagnosis**	
≤11.6	24 (22.6)
>11.6	82 (77.4)
**End-of-treatment PET SUV_{max}**	
≤5.4	116 (89.9)
>5.4	13 (10.1)
**CBC lymphocyte count**	
≤1.2.1×10^9/L	63 (64.3)
>1.2.1×10^9/L	35 (35.7)
**CBC lymphocyte/monocyte ratio**	
≤0.1	52 (53.0)
>0.1	46 (47.0)
**CD30 expression**	
Negative	38 (27.9)
Positive	98 (72.1)
**MUM1 expression**	
Negative	8 (15.4)
Positive	44 (84.6)
**Radiation therapy**	
No	21 (19.8)
Yes	85 (80.2)
**Stem cell transplant**	
No	135 (81.3)
Yes	31 (18.7)

PMBCL, primary mediastinal large B-cell lymphoma; LDH: lactate dehydrogenase; ECOG: Eastern Cooperative Oncology Group; IPI: International Prognostic Index; R-CHOP: rituximab, cyclophosphamide, doxorubicin, vincristine, and prednisone; R-EPOCH: rituximab, etoposide, prednisone, vincristine, cyclophosphamide, and doxorubicin; R-HCVAD: rituximab, cyclophosphamide, mesna, doxorubicin, vincristine, dexamethasone, methotrexate, and cytarabine; PET: positron emission tomography; SUV_{max}: maximum standardized uptake value; CBC: complete blood count

Supplemental Table 2. Comparison between patients who achieved and who did not achieve CR after the first line treatment

Characteristic	CR n (%)	Non-CR n (%)	*P*
**Age**			
≤60 years	115 (97.5)	48 (100)	0.56
>60 years	1 (2.5)	0 (0)	
**Sex**			
Female	72 (61.0)	22 (45.8)	0.085
Male	46 (39.0)	26 (54.2)	
**Stage**			
I-II	82 (70.7)	39 (81.2)	0.18
III-IV	34 (29.3)	9 (18.8)	
**B symptoms**			
No	79 (67.5)	21 (43.8)	**0.0053**
Yes	38 (32.5)	27 (56.3)	
**Serum LDH**			
Normal	35 (32.4)	10 (23.3)	0.33
Elevated	73 (67.6)	33 (76.7)	
**No. of extranodal sites**			
0-1	110 (94.8)	45 (93.7)	0.72
≥2	6 (5.17)	3 (6.3)	
**ECOG performance status**			
0-1	95 (81.9)	43 (91.5)	0.15
≥2	21 (18.1)	4 (8.5)	
**Mediastinal mass size**			
≤10 cm	53 (52.0)	17 (37.0)	0.117
>10 cm	49 (48.0)	29 (63.0)	
**Pleural effusion**			
No	113 (98.3)	45 (93.7)	0.11
Yes	2 (1.7)	3 (6.3)	
**IPI risk group**			
0-1	82 (73.2)	36 (78.3)	0.55
>1	30 (26.8)	10 (21.7)	
**Chemotherapy**			
R-CHOP	57(48.3)	32 (66.7)	**0.023***
R-EPOCH	44 (37.3)	11 (22.9)	
R-HCVAD	16 (13.6)	3 (6.3)	
Others	1 (0.8)	2 (4.2)	
**Ki-67 index**			
<70%	30 (46.9)	7 (28.0)	0.15
≥70%	34 (53.1)	18 (72.0)	
**CD30 expression**			
-	26 (27.1)	12 (30.0)	0.83
+	70 (72.9)	28 (70.0)	
**CD5 expression**			
-	52 (94.5)	26 (100)	0.55

+	3 (5.5)	0 (0)	
**CD23 expression**			
-	7 (22.6)	6 (40)	0.3
+	24 (77.4)	9 (60)	
**CD10 expression**			
-	74 (88.1)	26 (86.7)	1.0
+	10 (11.9)	4 (13.3)	
**MUM1 expression**			
-	7 (17.1)	1 (9.1)	1.0
+	34 (82.9)	10 (90.9)	
**BCL2 expression**			
-	5 (10.2)	3 (15.8)	0.68
+	44 (89.8)	16 (84.2)	
**BCL6 expression**			
-	3 (5.2)	5 (26.3)	**0.019**
+	55 (94.8)	14 (73.7)	
**CBC lymphocyte count**			
≤1.2.1×10^{9}/L	50 (64.9)	13 (61.9)	0.8
>1.2.1×10^{9}/L	27 (35.1)	8 (38.1)	
**CBC lymphocyte:monocyte ratio**			
≤0.1	40 (52.0)	12 (57.1)	0.81
>0.1	37 (48.1)	9 (42.9)	
**PET SUV_{max} at diagnosis**			
≤11.6	18 (22.5)	6 (23.1)	1.0
>11.6	62 (77.5)	20 (76.9)	
**End-of-treatment PET SUV_{max}**			
≤5.4	96 (100)	20 (60.6)	**<0.0001**
>5.4	0 (0)	13 (39.4)	

CR: complete response; LDH: lactate dehydrogenase; ECOG: Eastern Cooperative Oncology Group; IPI: International Prognostic Index; R-CHOP: rituximab, cyclophosphamide, doxorubicin, vincristine, and prednisone; R-EPOCH: rituximab, etoposide, prednisone, vincristine, cyclophosphamide, and doxorubicin; R-HCVAD: rituximab, cyclophosphamide, mesna, doxorubicin, vincristine, dexamethasone, methotrexate, and cytarabine; CBC: complete blood count; PET: positron emission tomography; SUV: standardized uptake value.

*R-CHOP vs. R-EPOCH plus R-HCVAD.

Significant *P* values are in bold.

Supplementary Table 3. Multivariate survival analysis for four significant factors adjusting for clinical parameters composing the IPI by univariate analysis

Variable	OS			PFS		
	HR	95% CI	*P*	HR	95% CI	*P*
**Ki-67 and clinical factors**						
Age >60 years	81.0	4.39-1495.4	**0.003**	17.89	1.81-176.36	**0.013**
Stage II-III	1.65	0.55-4.95	0.37	2.51	1.04-6.06	**0.04**
Elevated LDH	2.03	0.45-9.09	0.36	1.28	0.46-3.59	0.63
Extranodal sites >1	0.46	0.053-4.05	0.49	0.66	0.14-3.08	0.59
ECOG >1	<0.001	0-N/A	0.98	0.11	0.015-0.88	**0.037**
Ki-67 ≥70%	10.64	1.38-81.8	**0.023**	4.54	1.54-13.40	**0.006**
**MUM1 and clinical factors**						
Age >60 years	1.93	0.13-28.44	0.63	1.50	0.16-13.7	0.72
Stage II-III	2.67	0.32-22.26	0.36	5.88	1.34-25.72	**0.019**
Elevated LDH	421943.14	0-2.18E+265	0.97	5.69	0.64-50.68	0.12
Extranodal sites >1	<0.001	0-5.77E+277	0.97	<0.001	0-N/A	0.99
ECOG >1	<0.001	0-3.45E256	0.97	0.095	0.01-0.91	**0.041**
MUM1 positive	0.12	0.015-0.96	**0.045**	0.53	0.12-2.37	0.40
**CBC lymphocyte:monocyte ratio and clinical factors**						
Age >60 years	5.65	0.61-52.3	0.13	7.19	0.78-66.20	0.082
Stage II-III	2.36	0.51-10.82	0.27	5.88	1.34-25.72	**0.019**
Elevated LDH	518287.1	0-N/A	0.98	1.12	0.22-5.77	0.90
Extranodal sites >1	<0.001	0-N/A	0.99	<0.001	0-N/A	0.99
ECOG >1	0.24	0.025-2.20	0.21	0.25	0.047-1.32	0.10
Lymphocyte:monocyte ratio >11.6	0.64	0.15-2.74	0.55	0.24	0.06-0.99	**0.048**
**End-of-treatment PET SUV_{max} and clinical factors**						
Age >60 years	<0.001	0-N/A	1.0	<0.001	0-N/A	0.99
Stage II-III	3.48	1.03-11.7	**0.044**	2.84	1.10-7.35	**0.031**
Elevated LDH	4.02	0.49-32.95	0.19	2.32	0.64-8.39	0.20
Extranodal sites >1	<0.001	0-N/A	0.99	<0.001	0-N/A	0.98
ECOG >1	0.26	0.029-2.23	0.22	0.34	0.071-1.66	0.18
End-of-treatment PET SUV_{max} >5.4	3.40	0.90-12.78	0.071	6.21	2.24-17.17	**<0.001**

OS: overall survival; PFS: progression-free survival; HR, hazard ratio; LDH: lactate dehydrogenase; ECOG: Eastern Cooperative Oncology Group; CBC: complete blood count; PET: positron emission tomography; SUV_{max}: maximum standardized uptake value.

Significant *P* values are in bold.

Supplementary Table 4. Clinicopathologic characteristics of patient groups

Characteristic	End-of-treatment PET SUV ≤5.4 n (%)	End-of-treatment PET SUV >5.4 n (%)	*P*	No SCT n (%)	SCT n (%)	*P*
**Age**						
≤60 years	115 (99.1)	13 (100)	1.0	132 (97.8)	31 (100)	1.0
>60 years	1 (0.9)	0 (0)		3 (2.2)	0 (0)	
**Sex**						
Female	66 (56.9)	5 (38.5)	0.25	76 (56.3)	18 (58.1)	0.84
Male	50 (43.1)	8 (61.5)		59 (43.7)	13 (41.9)	
**Stage**						
I-II	85 (73.9)	9 (69.2)	0.74	101 (74.8)	20 (69)	0.50
III-IV	30 (26.1)	4 (30.8)		33 (25.2)	10 (31)	
**B symptoms**						
No	74 (64.3)	5 (38.5)	0.079	84 (62.7)	16 (51.6)	0.22
Yes	41 (35.7)	8 (61.5)		50 (37.3)	15 (48.4)	
**Serum LDH**						
Normal	33 (30.3)	1 (10)	0.28	39 (31.5)	6 (22.2)	0.24
Elevated	76 (69.7)	9 (90)		85 (68.5)	21 (77.8)	
**No. of extranodal sites**						
0-1	108 (93.9)	13 (100)	1.0	127 (94.8)	28 (93.3)	0.66
≥2	7 (6.1)	0 (0)		7 (5.2)	2 (6.7)	
**ECOG performance status**						
0-1	91 (80.5)	13 (100)	0.12	111 (83.5)	27 (90)	0.57
≥2	22 (19.5)	0 (0)		22 (16.5)	3 (10)	
**Mediastinal mass size**						
≤10 cm	52 (48.1)	5 (38.5)	0.57	60 (49.6)	10 (37)	0.29
>10 cm	56 (51.9)	8 (61.5)		61 (50.4)	17 (63)	
**IPI risk group**						
0-1	85 (75.9)	7 (58.3)	0.29	99 (76.2)	19 (67.9)	0.33
>1	27 (24.1)	5 (41.7)		31 (23.8)	9 (32.1)	
**Chemotherapy**						
R-CHOP	53 (45.7)	8 (61.5)	0.38*	70 (51.9)	19 (63.3)	0.42*
R-EPOCH	44 (37.9)	5 (38.5)		46 (34.1)	9 (30)	
R-HCVAD	17 (14.7)	0 (0)		17 (12.9)	2 (6.7)	
Others	2 (1.7)	0 (0)		2 (1.5)	0 (0)	
**Therapy response**						
CR	96 (83.6)	0 (0)	**<0.0001[#]**	107 (79.3)	11 (35.5)	**<0.0001[#]**
PR	19 (15.5)	9 (69.2)		23 (17)	16 (51.6)	
SD	0 (0)	1 (7.7)		2 (1.5)	0 (0)	
PD	1 (0.9)	3 (23.1)		3 (2.2)	4 (12.9)	

PET: positron emission tomography; SUV: standardized uptake value; SCT: hematopoietic stem cell transplantation; LDH: lactate dehydrogenase; ECOG: Eastern Cooperative Oncology Group; IPI: International Prognostic Index; R-CHOP: rituximab, cyclophosphamide, doxorubicin, vincristine, and prednisone; R-EPOCH: rituximab, etoposide, prednisone, vincristine, cyclophosphamide, and doxorubicin; R-HCVAD: rituximab, cyclophosphamide, mesna, doxorubicin, vincristine, dexamethasone, methotrexate, and cytarabine; CR: complete response; PR: partial response; SD: stable disease; PD: progressive disease. *R-CHOP vs. R-EPOCH and R-HCVAD. [#]CR vs. non-CR. Significant *P* values are in bold.
