# Supplementary material for: Prognostic factors, therapeutic approaches, and distinct immunobiologic features in patients with primary mediastinal large B-cell lymphoma on long-term follow-up
Source: Blood Cancer J. 2020 May 4;10(5):49. doi: 10.1038/s41408-020-0312-7 (PMC7198569; doi:10.1038/s41408-020-0312-7)

Suppl. Figure 1. Prognostic factors at diagnosis. (A) Elderly age was associated with significantly poorer overall survival. Advanced stage and elevated LDH levels showed nonsignificant trends toward poorer overall survival. **(B)** Low PET SUV_{max}, CD30 positivity, and high absolute lymphocyte counts showed nonsignificant trends toward favorable impact on progression-free survival.

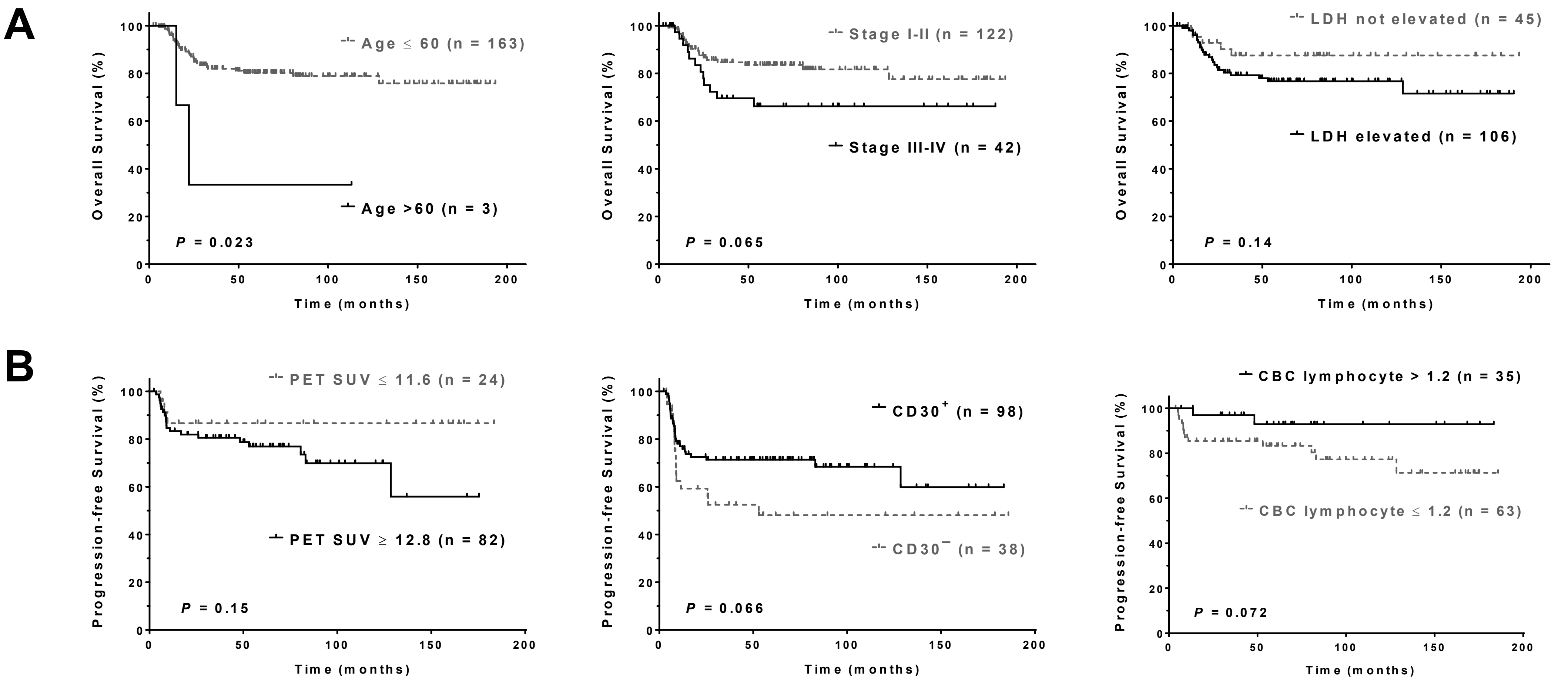

Suppl. Figure 2. Treatment options and prognosis. (A) Radiation therapy as consolidation did not have a significant effect on survival in PMBCL. **(B)** In relapsed/refractory PMBCL patients, radiation therapy after salvage therapy did not have a significant effect on survival.

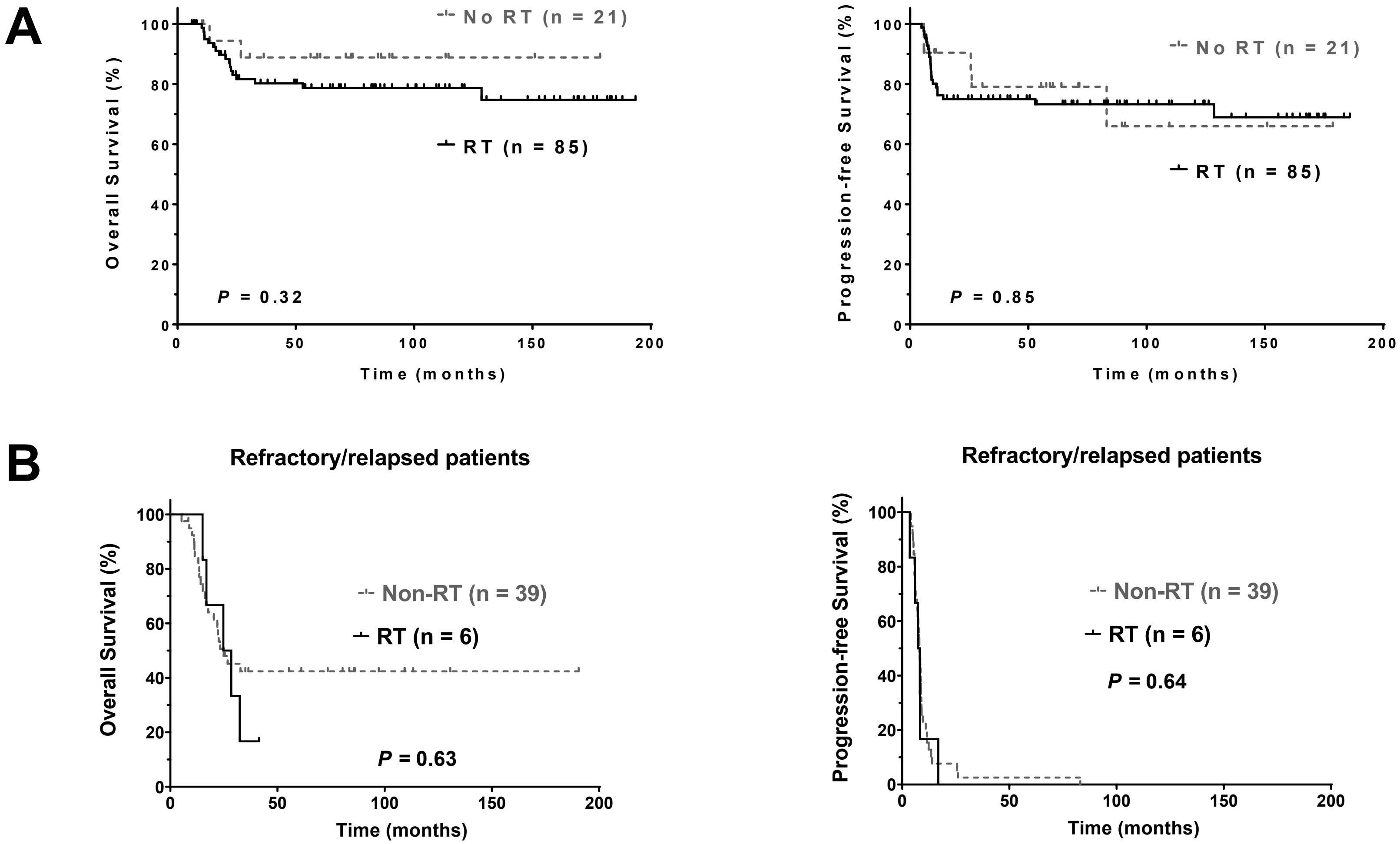

Suppl. Figure 3. Treatment options and prognosis. (A) Stem cell transplant improved overall survival in patients with relapsed/refractory PMBCL regardless of frontline treatment regimens. **(B)** Stem cell transplant did not show survival benefit in PMBCL patients without relapse after chemotherapy with a CR/PR.

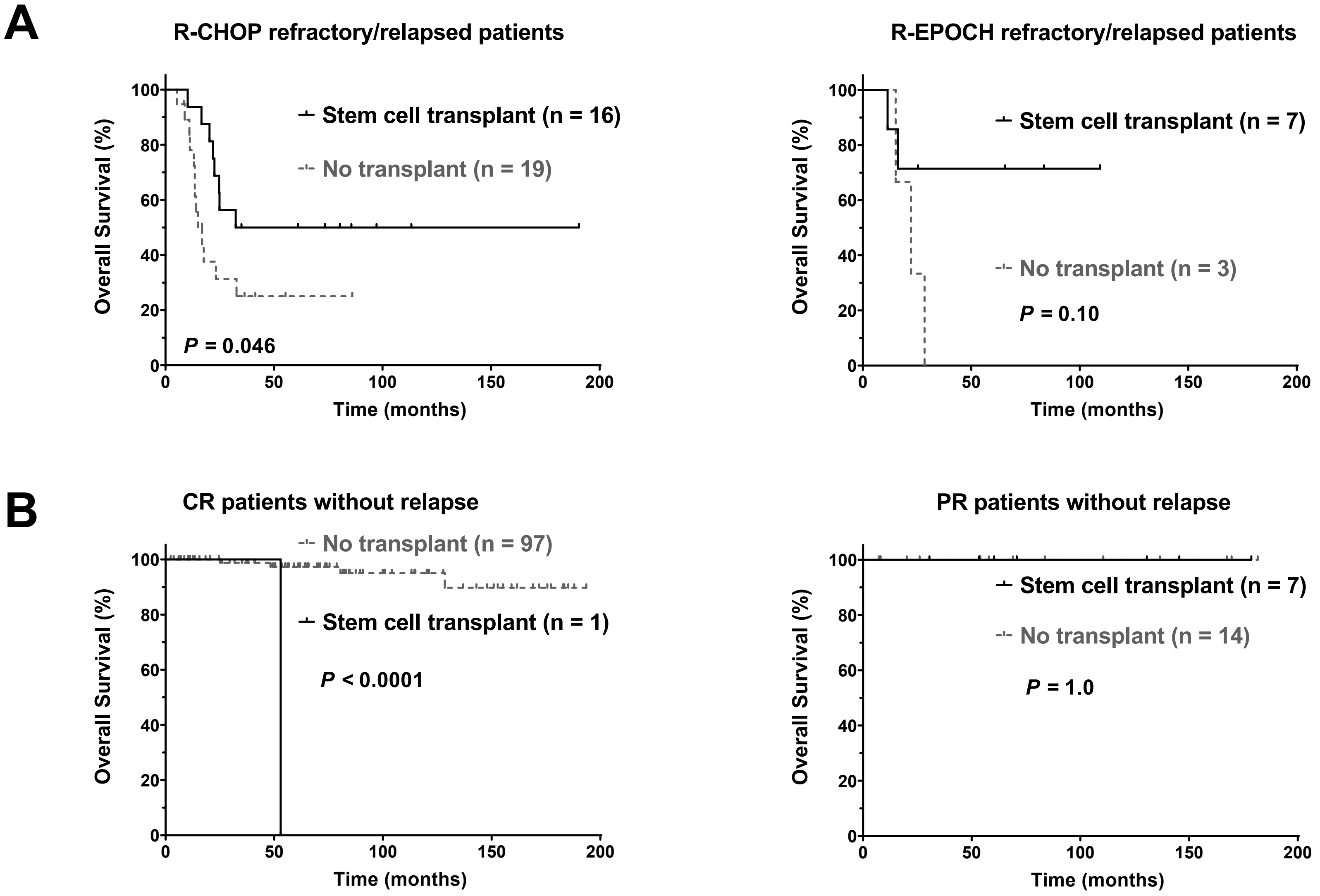

Suppl. Figure 4. Additional survival curves for CTLA-4 expression in T cells, PD-1 expression in T cells, and PD-L2 expression in B cells in PMBCL.

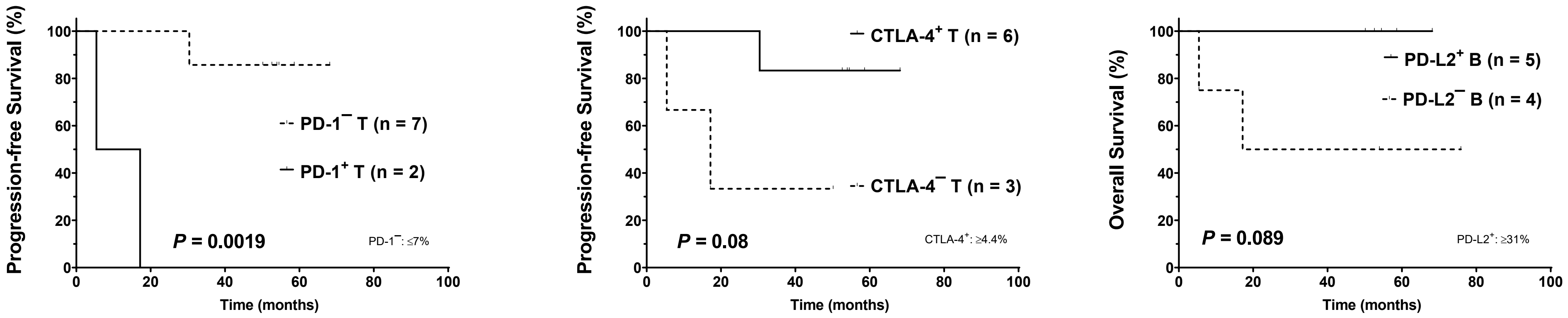

Suppl. Figure 5. Significantly differentially expressed genes between PMBCL and DLBCL NOS

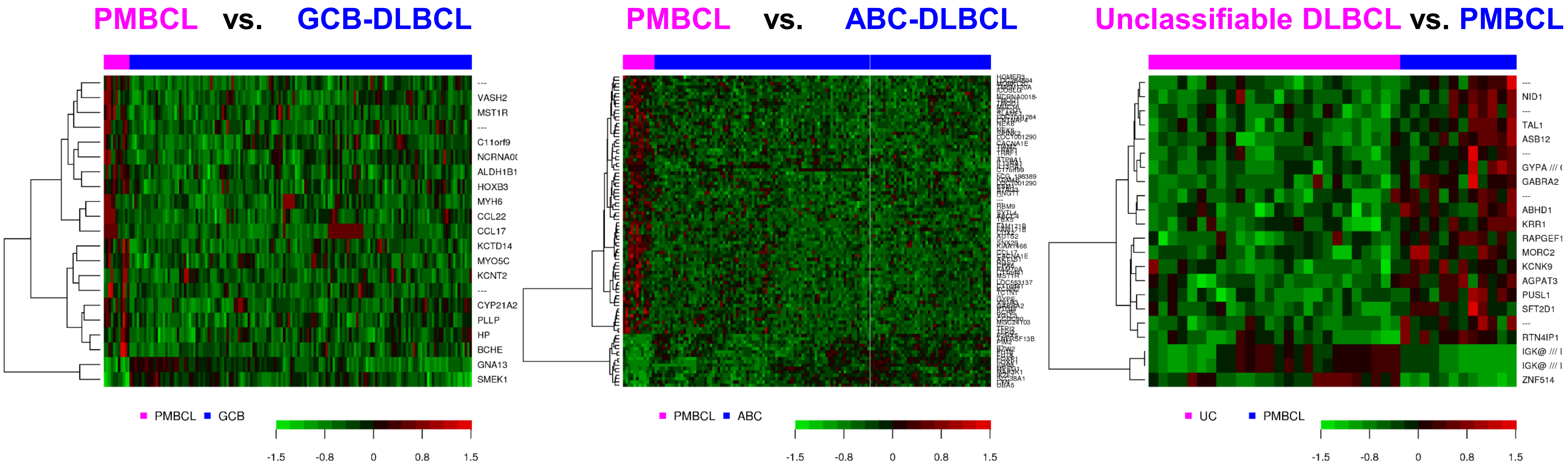

Suppl. Figure 6. miRNA profile for PMBCL. (A) Heatmap for top 10 upregulated or downregulated miRNAs in dead versus alive PMBCL patients. **(B)** Heatmap for 25 downregulated and 20 upregulated miRNAs in PMBCL patients with progression/relapse events compared with those without progression/relapse events.

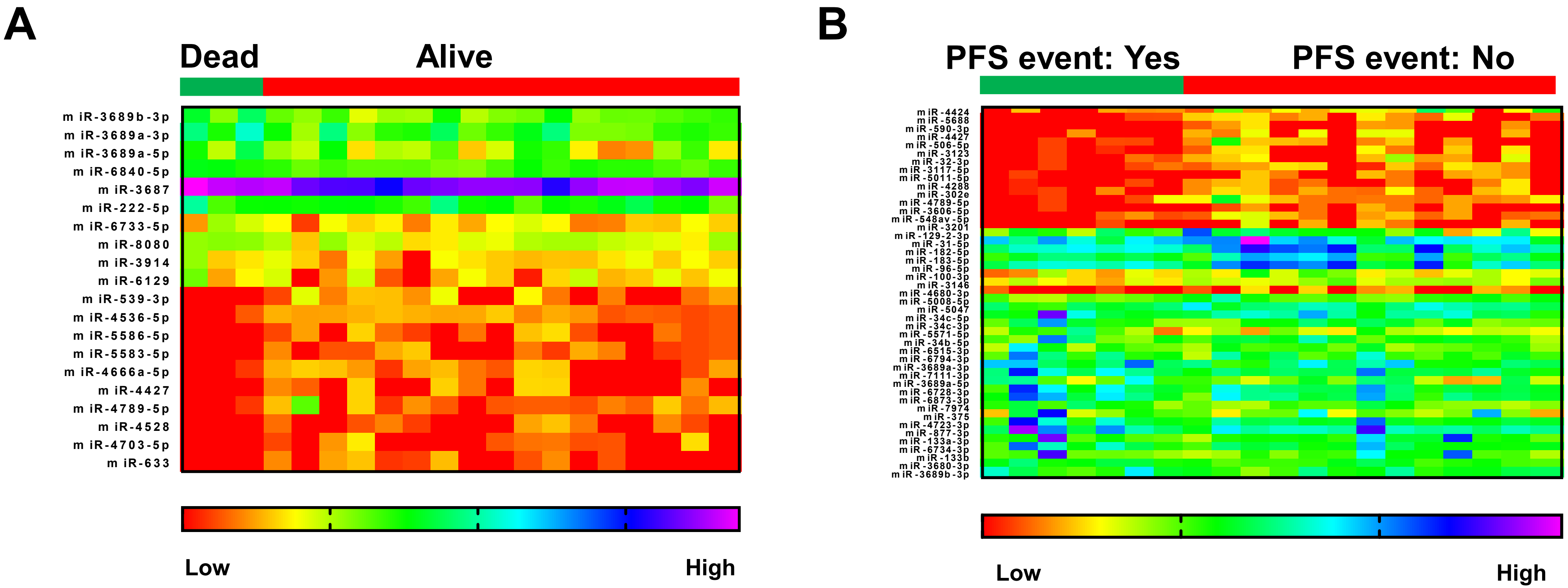

Supplement: Supplementary file 2 — Supplemental Figures [file 41408_2020_312_MOESM2_ESM.pdf]
